# Realizing the Application of EEG Modeling in BCI Classification: Based on a Conditional GAN Converter

**DOI:** 10.3389/fnins.2021.727394

**Published:** 2021-11-11

**Authors:** Xiaodong Zhang, Zhufeng Lu, Teng Zhang, Hanzhe Li, Yachun Wang, Qing Tao

**Affiliations:** ^1^School of Mechanical Engineering, Xi’an Jiaotong University, Xi’an, China; ^2^Shaanxi Key Laboratory of Intelligent Robot, Xi’an Jiaotong University, Xi’an, China; ^3^School of Mechanical Engineering, Xinjiang University, Wulumuqi, China

**Keywords:** BCI, EEG, GAN, modeling, simulation, classification

## Abstract

Electroencephalogram (EEG) modeling in brain-computer interface (BCI) provides a theoretical foundation for its development. However, limited by the lack of guidelines in model parameter selection and the inability to obtain personal tissue information in practice, EEG modeling in BCI is mainly focused on the theoretical qualitative level which shows a gap between the theory and its application. Based on such problems, this work combined the surface EEG simulation with a converter based on the generative adversarial network (GAN), to establish the connection from simulated EEG to its application in BCI classification. For the scalp EEGs modeling, a mathematical model was built according to the physics of surface EEG, which consisted of the parallel 3-population neural mass model, the equivalent dipole, and the forward computation. For application, a converter based on the conditional GAN was designed, to transfer the simulated theoretical-only EEG to its practical version, in the lack of individual bio-information. To verify the feasibility, based on the latest microexpression-assisted BCI paradigm proposed by our group, the converted simulated EEGs were used in the training of BCI classifiers. The results indicated that, compared with training with insufficient real data, by adding the simulated EEGs, the overall performance showed a significant improvement (*P* = 0.04 < 0.05), and the test performance can be improved by 2.17% ± 4.23, in which the largest increase was up to 12.60% ± 1.81. Through this work, the link from theoretical EEG simulation to BCI classification has been initially established, providing an enhanced novel solution for the application of EEG modeling in BCI.

## Introduction

Non-invasive brain-computer interface (BCI) as a branch in neuroscience, has continuously been a hotspot in recent decades. The focal point of the research has been shifted from the signal detection at first (Berger, 1929), to the paradigm proposal (Herrmann, 2001; Pfurtscheller and Neuper, 2001; Wolpaw et al., 2002; Polich, 2007), then the EEG decoding and classification (Koelstra et al., 2012; Chen et al., 2015; Muller-Putz et al., 2016). With the growing publication of related studies, attention began to be paid to the generation mechanism and modelling of EEG in BCI, attempting to provide more solid theoretical foundations.

Unlike clinical medicine (Sanz-Leon et al., 2015), EEG modeling launched by BCI usually adopted the neuronal population level-based modeling approach and has been more concerned with the theoretical qualitative simulation. The “neural population” had first been put forward in the 1970s, assuming that all nervous processes can be dependent upon the interaction of excitatory and inhibitory cells (Freeman, 1972, 1973, 1975; Wilson and Cowan, 1972, 1973). Later, it was adopted by Lopes da Silva in modeling the generation of rhythmic activity (Lopesdas et al., 1974; Lopes da Silva et al., 1976). In 1995, the neural mass model (NMM) was proposed by Jansen and Rit (1995) in the mathematical computation of visual evoked potential (Jansen et al., 1993). Since then, Jasen-Rit NMM established the connection between deep EEG modeling and the BCI paradigm, and became popular in terms of BCI-related EEG modeling. In 2006, Wendling NMM (Wendling et al., 2002), as an improved Jasen-Rit, was utilized in the simulation of cortical activity during motor tasks (Zavaglia et al., 2006). Afterward, NMM was applied in the EEG simulation based on the scene graph steady-state visual evoked potentials BCI paradigm (Li et al., 2018a) and the EEG driven by facial expression (Zhang et al., 2016).

Neural mass model-based EEG modeling does evidence the signal response under the BCI paradigm to some extent, but by far it has not formed guidance for the paradigm design and it is still a certain distance away from application in BCI. Firstly, according to the anatomy, the output of NMM is the postsynaptic membrane potential, which can be regarded as the deep source of surface EEG, but not the collected one. Certainly, under well-selected parameters, phenomena in surface EEG can be reproduced (i.e., significant frequency distribution (Jansen and Rit, 1995), rhythmic activity (Ursino et al., 2010), energy undulation (Zavaglia et al., 2006)); However, the selection of NMM parameters in BCI relied largely on manual adjustment and consumed large efforts. Another challenge is the forward computation from the inner source to the scalp signal, which especially requires one’s tissue conductivity and geometry (Cosandier-Rimele et al., 2010). Although the simplified three-shell concentric spherical head model can be adopted (Berg and Scherg, 1994), without extra modification the result still stayed at the theoretical level (meanwhile, it is unaffordable and impossible for investigators to obtain complex tissue data from every user in practice). Obstacles above (i.e., the selection of NMM parameters, and the lack of individual biometric data) limited the application of EEG simulation in BCI. However, without the application, modeling, and simulation of EEG, it lacked practical meaning, was stuck in the theoretical level, and felt within an inch of enough.

In the other aspect, building the link between EEG simulation and its application in BCI is also valuable for BCI. Simulated EEG has its natural advantage in data amount. If the simulated signal can be applied to BCI training, it can become another feasible solution to reduce the amount of pre-collected training data and alleviate the problem of data insufficiency. To build such a link, the core problem lies in how to realize the personal scalp EEG simulation without detailed individual tissue data and the fine tuning of the NMM parameter. Encouraged by the work of probabilistic forecasting (Koochali et al., 2019), data augmentation (Fan et al., 2020; Luo et al., 2020), and EEG feature generating (Krishna et al., 2021) with generative adversarial networks (GAN), in this work, we proposed an EEG simulation method with conditional GAN combined to compensate for the lack of tissue information, hence establishing the link between EEG modeling and its application.

In this paper, emphasis was laid on surface EEG modeling and realizing its application in BCI classification. In the methodology section, the generation physics of surface EEG were explained firstly thus deriving the mathematical model of scalp EEG; Then, focusing on the realistic dilemma, a conditional GAN converter was proposed to transform the simulated EEGs from the theoretical-only to its practical version, to overcome the lack of individual bio-information. In the data acquisition section, the experimental EEGs acquisition and the paradigm-related parameters were stated. In the results section, the feasibility of applying the EEG simulation to the BCI classification was estimated. As followed, the discussion section discussed the performance and the limitation of this work, and the conclusion was summarized in the conclusion section.

## Methodology

**FIGURE 1:**
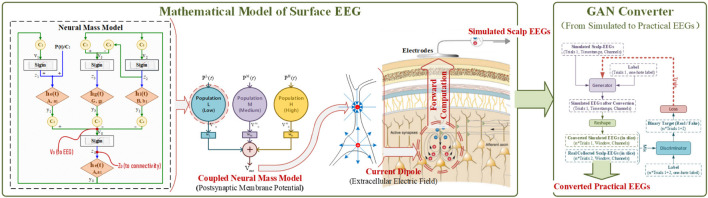
The schematics diagram of methodology.

To build the connection between EEG modeling and its application in BCI, the method started from the scalp EEG modeling, and then a converter based on the conditional GAN was combined to transfer the theoretical-only simulated-EEG to its practical version. The overall schematics diagram is illustrated in Figure 1.

### The Mathematical Model of Surface Electroencephalogram

**FIGURE 2:**
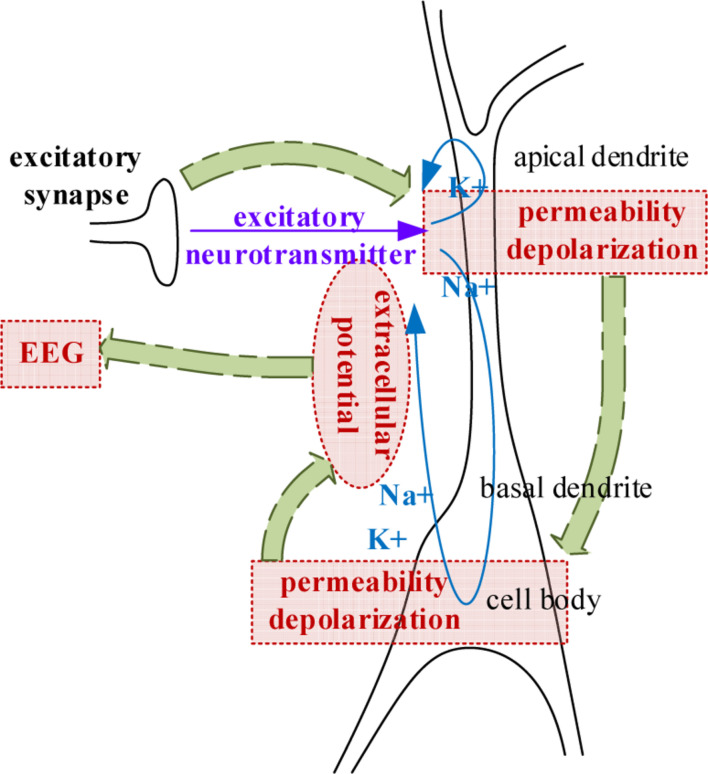
The generation of extracellular potentials.

Electroencephalogram measures the large-scale simultaneous activation of the brain neurons through the surface electrodes placed on the scalp (Berger, 1929). It is generally believed that the extracellular potential field generated by postsynaptic potentials is the source of an EEG (Baillet et al., 2001; Hallez et al., 2007). Since only the regular arrangements of neurons can amplify their extracellular potential field to a measurable extent, the extracellular potential fields raised by the postsynaptic potential of pyramidal cells are generally admitted to be the generator of the EEG. The extracellular potential is caused by the migration of positively charged ions (Schaul, 1998), as illustrated in Figure 2. From the extracellular environment to the brain surface, the spread pathway contains the gray matter, the white matter, the cerebrospinal fluid (CSF), the skull, and the scalp. Only by passing through various tissues, can the electrical activity deep within the brain be finally detected by EEG electrodes. According to the physics of surface EEG, the modeling process of scalp EEG can be divided into three nodes: the postsynaptic membrane potential, the extracellular electric field and the forward computation from deep EEG to scalp EEG. Following are the details.

#### Modeling of Postsynaptic Membrane Potential

According to the physics, the modeling starts from the postsynaptic membrane potential. For being generated by large-scaled simultaneous activation, instead of isolated neurons, the cortical activity is simulated with the population-level based model: NMM.

**FIGURE 3:**
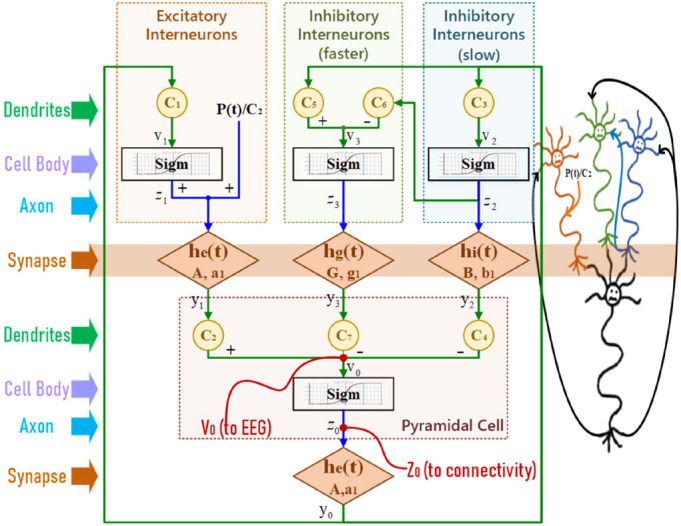
The structure of a single population model.

In NMM, one population represents neurons that are lumped together with the same membrane potential. A single population includes the interaction among four neural subgroups (Wendling et al., 2002), pyramidal cells, excitatory interneurons, inhibitory interneurons with slow synaptic kinetics, and inhibitory interneurons with faster synaptic kinetics, as illustrated in Figure 3.

In Figure 3, a static nonlinearity sigmoid relationship is used to convert the average postsynaptic membrane potentials to an average spike density. Then a second-order transfer function _*h*(*t*)_ transfers the presynaptic spike density to the postsynaptic membrane potential. This second-order representation of synaptic effect was firstly derived by Rotterdam (van Rotterdam et al., 1982) and is still in use. After simplification, the mathematical equations of Wendling’s NMM (Wendling et al., 2002) are


(1) $$\left\{ \begin{array}{c} {\ddot{y}}_{0}\left(t\right)=AaSigm\left[C_{2}y_{1}\left(t\right)-C_{4}y_{2}\left(t\right)-C_{7}y_{3}\left(t\right)\right]-2a{\dot{y}}_{0}\left(t\right)-a^{2}y_{0}\left(t\right)\\ {\ddot{y}}_{1}\left(t\right)=Aa\left(Sigm\left[C_{1}y_{0}\left(t\right)\right]+P\left(t\right)/C_{2}\right)-2a{\dot{y}}_{1}\left(t\right)-a^{2}y_{1}\left(t\right)\\ {\ddot{y}}_{2}\left(t\right)=BbSigm\left[C_{3}y_{0}\left(t\right)\right]-2b{\dot{y}}_{2}\left(t\right)-b^{2}y_{2}\left(t\right)\\ {\ddot{y}}_{3}\left(t\right)=GgSigm\left[C_{5}y_{0}\left(t\right)-C_{6}Sigm\left[C_{3}y_{0}\left(t\right)\right]\right]-2g{\dot{y}}_{3}\left(t\right)-g^{2}y_{3}\left(t\right) \end{array}\right.$$

where *A, B*, & *G* are the average gain in synaptic effect, and *a, b*, & *g* are the time constant; C_1_∼C_7_ represent the connectivity constants between neuron groups; P(*t*) is the sum of exogenous contributions. The sigmoid function is


(2) $$Sigm\left(v\right)=2e_{0}/\left(1+e^{r\left(s_{0}-v\right)}\right)$$

where *e*_0_ determines the maximum firing rate of the neural population, *s*_0_ is the mean firing threshold, and *r* is the slope.

**FIGURE 4:**
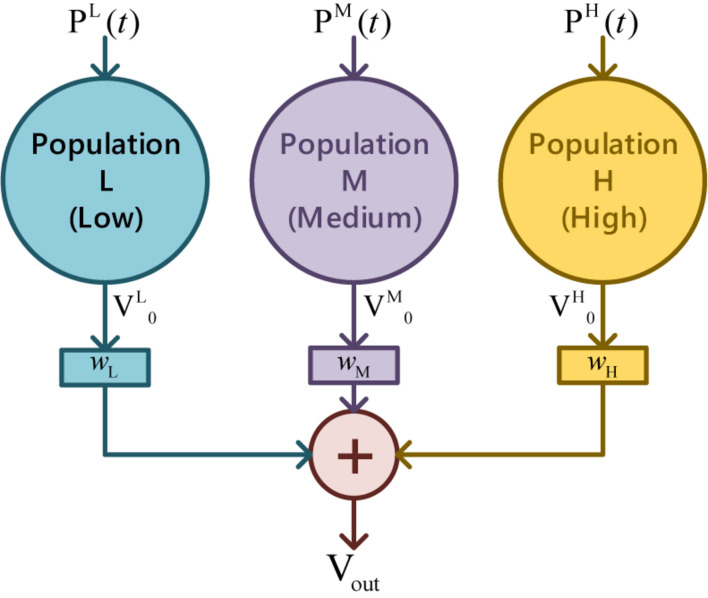
Parallel feedforward schema of triple populations.

Since a single population can only produce a unimodal spectrum, multiple coupled neural populations with different ways of connecting were studied (Wendling et al., 2000) to describe the neural activity in a wide bandwidth. By separating typical EEG into three bands as the low (4-12 Hz), the medium (12-30 Hz), and the high (>30 Hz) (Zavaglia et al., 2006), parallel feedforward schema of three populations were arranged to mimic the overall complexity of EEG, as illustrated in Figure 4. The postsynaptic membrane potential V_*out*_ under multi-parallel-populations is


(3) Vout=∑k=L,M,HwkVk(t)o

where superscript L, M, and H indicate the three populations in representative EEG bands, and *w*_*k*_(*k* = L, M, H) stands for the weights.

#### Equivalent Model of Extracellular Electric Field

**FIGURE 5:**
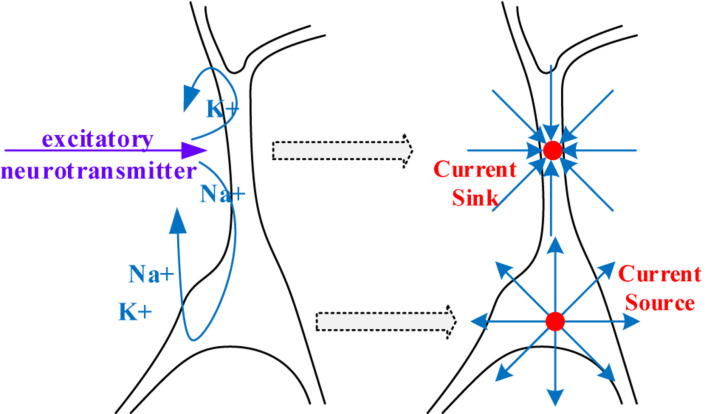
A simplified dipole model for generating the extracellular electric field.

The generator of the extracellular electric field can be viewed as a simplified electric model with two current monopoles, as demonstrated in Figure 5. At the apical dendrite side, a current sink is used to describe the influx of positive ions. At the cell body, a current source is placed to represent the injection of positively charged ions into the extracellular environment.

The frequency range and time-variation of measured EEGs indicate that there is no charge piled up in the conducting extracellular volume (Plonsey and Heppner, 1967). Thereby the simplified electric model can be regarded as a current dipole that consists of two equal anisotropic charges. Following the population level-based modeling approach in “Modeling of Postsynaptic Membrane Potential,” the simultaneous electrically active pyramidal cells in a small patch can be represented as one equivalent dipole (He et al., 2002).

The electric-field intensity of the dipole in a quasi-static field can be driven utilizing the gradient operator


**FIGURE 6:**
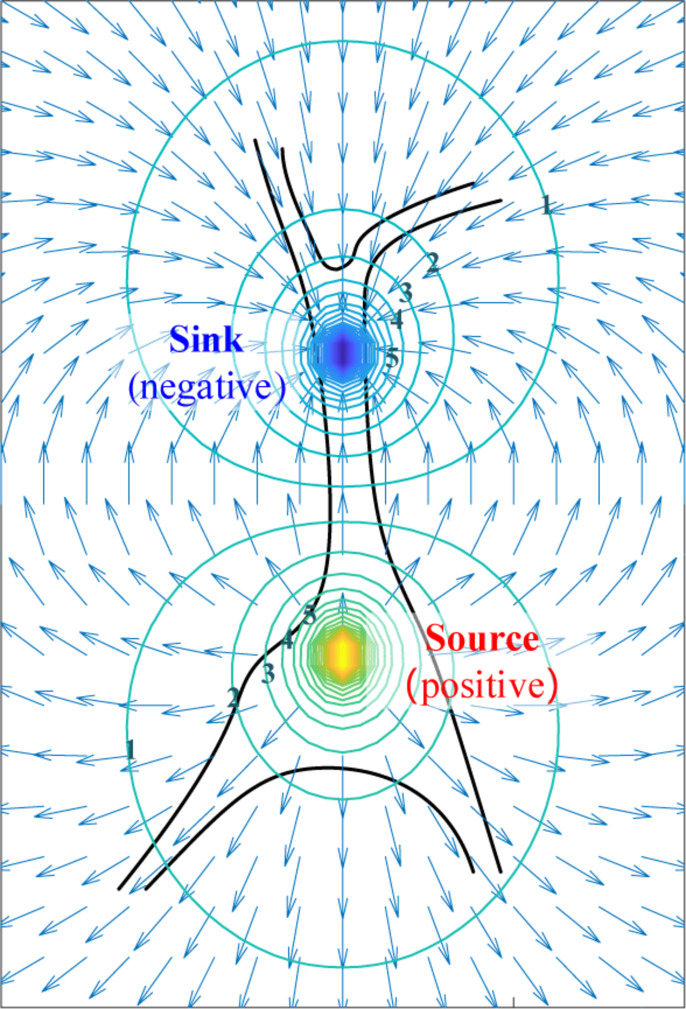
The simplified extracellular electric field and its equipotential lines generated by dipole.

(4) E=-∇⁡U

where **E** is the vector electric-field intensity in V/m, ∇ is the gradient operator, and U is the scalar potential field. For a dipole, the scalar potential in a vacuum at any point *a* is


(5) $${\text{U}}_{a}=k\frac{I\left(r_{2}-r_{1}\right)}{r_{1}r_{2}}\approx k\frac{\text{d}\cos\theta }{r^{2}}=k\frac{I\cdot p\cos\theta }{r^{2}}$$

where *k* is the scale factor, *r*_1_ *r*_2_ *r* are distances apart from the positive, the negative, and the dipole center respectively, *p* is distance between two monopoles, *I* is the current injected or removed, and **d** is the dipole moment. By combining (4) and (5), the equipotential lines and electric-field intensity in vicinity is illustrated in Figure 6.

#### Forward Computation of Surface Electroencephalogram

From the extracellular field to the scalp, charges pass through the gray matter, the white matter, the cerebrospinal fluid (CSF), the skull, and the scalp. Tissues on this pathway have their unique conductivities, resulting in a smearing effect on the forward potential computation (Wolters et al., 2006). Among them, the conductivities for gray matter, scalp, and CSF are isotropic (Baumann et al., 1997); While the white matter and the skull have anisotropic conductivity (Rush, 1900; Nicholson, 1965).

By multiplying the electric field *E* and the conductivity, the current density is introduced to describe the total flow of charge per time over a cross-section of area. According to Ohm’s law, the current density is


(6) J=κE

where vector field **J** in A/m^2^ indicates the current density perpendicular to the cross-sectional area of the conductor, and κ is the electrolytic conductivity with units S/m. For isotropic conductivities κ is a scalar; While for anisotropic conductivities, κ ∈ *ℝ*^3×3^ is a position dependent conductivity tensor as


(7) $$\kappa =\left[\begin{array}{ccc} \kappa_{11} & \kappa_{12} & \kappa_{13}\\ \kappa_{12} & \kappa_{22} & \kappa_{23}\\ \kappa_{13} & \kappa_{23} & \kappa_{33} \end{array}\right]$$

By applying the divergence operator to the current density, according to Poisson’s equation, relationships can be defined


(8) $$\nabla \cdot J=\lim_{G\to 0}\frac{1}{G}\oint_{\partial G}\text{J}d\text{S}=I_{m}$$

where the ∇ ⋅ ***J*** is often called the current source density and symbolized with *I*_*m*_ in Enderle (2012). To compute the potential field on different tissues, combing equation (4), (6), and (8), a general formed Poisson’s differential equation can be obtained


(9) $$-\nabla \cdot \left(\kappa \nabla \left(\text{U}\right)\right)=I_{m}$$

For a dipole, the current source density at any point can be written as a delta function with its distance from the positive and the negative.


(10) $$\nabla \cdot \text{J}=I\delta \left(r_{1}\right)-I\delta \left(r_{2}\right)=-\nabla \cdot \left(\kappa \nabla \left(\text{U}\right)\right)$$

where *r*_1_ *r*_2_ are the same as in (5).

In the Cartesian coordinate system, setting the positive monopole at position (*x*_1_, *y*_1_, *z*_1_) and the negative at (*x*_2_, *y*_2_, *z*_2_), at any point (*x*, *y*, *z*) (10) becomes


(11) $$\begin{array}{cc} \kappa_{11}\frac{\partial^{2}\text{U}}{\partial x^{2}}+\kappa_{22}\frac{\partial^{2}\text{U}}{\partial y^{2}}+\kappa_{33}\frac{\partial^{2}\text{U}}{\partial z^{2}}+2\left(\kappa_{12}\frac{\partial^{2}\text{U}}{\partial x\partial y}+\kappa_{13}\frac{\partial^{2}\text{U}}{\partial x\partial z}+\kappa_{23}\frac{\partial^{2}\text{U}}{\partial y\partial z}\right) & \\ +\left(\frac{\partial \kappa_{11}}{\partial x}+\frac{\partial \kappa_{12}}{\partial y}+\frac{\partial \kappa_{13}}{\partial z}\right)\frac{\partial \text{U}}{\partial x}+\left(\frac{\partial \kappa_{12}}{\partial }+\frac{\partial \kappa_{22}}{\partial y}+\frac{\partial \kappa_{23}}{\partial z}\right)\frac{\partial \text{U}}{\partial y}+(\frac{\partial \kappa_{13}}{\partial x} & \\ +\frac{\partial \kappa_{23}}{\partial y}+\frac{\partial \kappa_{33}}{\partial z})\frac{\partial \text{U}}{\partial z}=-I\delta (x-x_{1})\delta (y-y_{1})\delta (z-z_{1})+ & \\ I\delta \left(x-x_{2}\right)\delta \left(y-y_{2}\right)\delta \left(z-z_{2}\right) & \end{array}$$

for anisotropic conductivities. For isotropic conductivities with a scalar κ, (10) can be written as


(12) $$\begin{array}{c} \frac{\partial }{\partial x}\left(\kappa \frac{\partial \text{U}}{\partial x}\right)+\frac{\partial }{\partial y}\left(\kappa \frac{\partial \text{U}}{\partial y}\right)+\frac{\partial }{\partial z}\left(\kappa \frac{\partial \text{U}}{\partial z}\right)=-I\delta \left(x-x_{1}\right)\delta \left(y-y_{1}\right)\\ \delta \left(z-z_{1}\right)+I\delta \left(x-x_{2}\right)\delta \left(y-y_{2}\right)\delta \left(z-z_{2}\right) \end{array}$$

### GAN Converter: From Simulated Signal to Practical Electroencephalograms

To accurately simulate the scalp EEGs, one’s unique tissue conductivity, geometry, and the location of the EEG generator are crucial in the forward computation. Such data have to be measured with specific equipment (e.g., Magnetic Resonance Imaging). But in practice, for BCI, it is impossible to get this biological information from every user. Without detailed personal data, however, the simulated scalp EEGs can only stop at theory without being used in realistic applications. To mimic one’s real EEGs in the lack of individual bio-information, the generative adversarial network (GAN) was adopted to convert the simulated signal into its practical version.

#### Conditional Generative Adversarial Network for Practical Electroencephalograms Imitation

Generative adversarial network was firstly proposed by Goodfellow et al. (2014), which provides the ability to counterfeit images that are statistically indistinguishable from real ones. A GAN consists of two networks: a Generator and a Discriminator. The Generator is trained to produce counterfeits that can deceive the Discriminator. As an adversary, the Discriminator aims at distinguishing the true from the fake. Inspired by GAN’s genius in image forgery, it was adopted in this work as a powerful converter to transform the simulated scalp EEGs from the theoretical-only to the practical.

To transform the simulated EEGs hence applying in the training of BCI classifier, conditional GAN was adopted to output signals with labels as the specified intention. For traditional GANs, the input of the Generator is a random vector, and several up-sampling computations are used to expand pixels. However, as a converter in this research, the simulated scalp EEGs were fed as input instead, without extra up-sampling.

**FIGURE 7:**
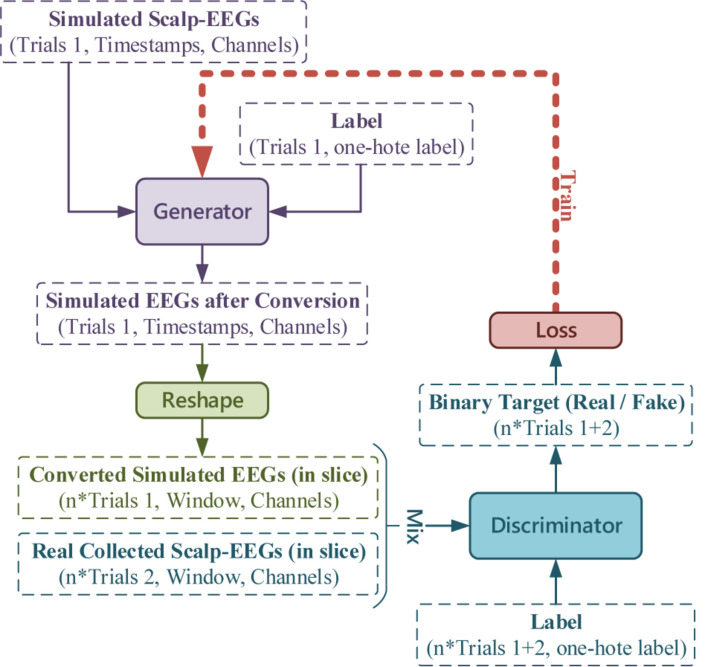
The conditional GAN for simulated EEGs conversion, where the number of trials, timestamps, channels, and labels, and the window length is in accordance with the experimental setup (latterly stated in RESULTS).

According to our previous experimental setup in the BCI serial studies (Lu et al., 2018a, 2020; Zhang et al., 2021b), to realize the real-time decoding, continuous collected EEGs (4 s) were sliced into short-windowed (100 ms) to form the training-set. To reproduce the dataset forming procedure and augment the real EEGs samples, the output of the Generator was sliced to window-length size before entering the Discriminator. The conditional GAN in this work is illustrated in Figure 7.

#### Generator and Discriminator

**FIGURE 8:**
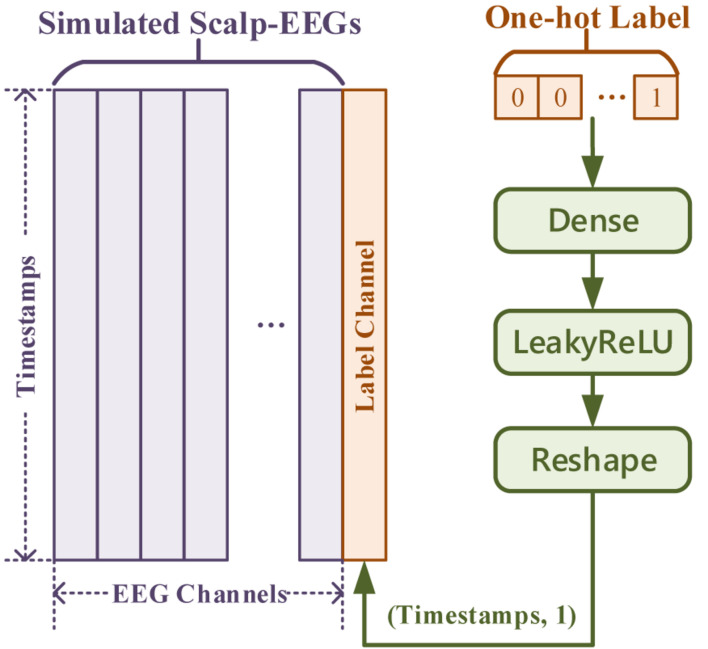
The label input in “simulated EEGs conversion GAN.”

In general cases, the input for the Generator is one-dimensional noise. Thus, one-hot labels for conditional GAN can be concatenated just after the noise. But in this work, with inputs being multi-channeled simulated scalp EEGs, one-hot labels were fed and transformed as an extra channel concatenated with the simulated signal. The transform of the label input is demonstrated in Figure 8, of which the Generator and the Discriminator shared the same method but a different number of dense nodes.

The detailed design of the Generator and the Discriminator is listed in Tables 1, 2. In both networks, the selection of kernel size followed the principle from a rough glance to a fine adjustment. The optimizer and the cost function of GAN are shown in Table 3.

**TABLE 1:** Summary of the generator.

Layer	Method	Parameter	Value
InputLayer_1	Label input	Shape	(None, One-hot label)
Dense_1	—	Units	Timestamps
		Activation	LeakyReLU
Reshape_1	—	Output	(None, Timestamps, 1)
InputLayer_2	Simulated Scalp EEGs input	Shape	(None, Timestamps, Channels)
Concatenate_1	Reshape_1 & InputLayer_2	Axis	2
Conv1D_1	Temporal dimension	Filters	64
		Kernel Size	64
		Stride	1
		Padding	Same
		Activation	LeakyReLU
Conv1D_2	Temporal dimension	Filters	52
		Kernel Size	32
		Stride	1
		Padding	Same
		Activation	LeakyReLU
Conv1D_3	Temporal dimension	Filters	42
		Kernel Size	16
		Stride	1
		Padding	Same
		Activation	LeakyReLU
Conv1D_4	Temporal dimension	Filters	36
		Kernel Size	6
		Stride	1
		Padding	Same
		Activation	LeakyReLU
Conv1D_5	Temporal dimension	Filters	Channels
		Kernel Size	2
		Stride	1
		Padding	Same
		Activation	Tanh

**TABLE 2:** Summary of the discriminator.

Layer	Method	Parameter	Value
InputLayer_1	Label input	Shape	(None, One-hot label)
Dense_1	—	Units	Window
		Activation	LeakyReLU
Reshape_1	—	Output	(None, Window, 1)
InputLayer_2	Mixed EEGs input	Shape	(None, Window, Channels)
Concatenate_1	Reshape_1 & InputLayer_2	Axis	2
Conv1D_1	Temporal dimension	Filters	16
		Kernel Size	32
		Stride	1
		Activation	LeakyReLU
Conv1D_2	Temporal dimension	Filters	8
		Kernel Size	16
		Stride	1
		Activation	LeakyReLU
Conv1D_3	Temporal dimension	Filters	4
		Kernel Size	8
		Stride	1
		Activation	LeakyReLU
Conv1D_4	Temporal dimension	Filters	2
		Kernel Size	4
		Stride	1
		Activation	LeakyReLU
Flatten_1	—	—	—
Dropout_1	—	Value	0.4
Dense_2	—	Units	1
		Activation	sigmoid
Loss Function	Binary Cross entropy	—	—
Optimizer	RMSprop	Learning rate	0.0008
		Clip value	1.0
		Decay	1e-8
		Activation	Tanh

**TABLE 3:** Optimizer and cost of GAN.

Type	Method	Parameter	Value
Loss Function	Binary Cross entropy	—	—
Iteration	—	—	200
Optimizer	RMSprop	Learning rate	0.0004
		Clip value	1.0
		Decay	1e-8

In the training process of GAN, the output of the Generator was unclear at the beginning. Then the awkward outputs mixed with the real collected EEGs were fed into the Discriminator to train a pair of discerning eyes. Subsequently, the Discriminator was frozen, and the Generator was trained to confuse the Discriminator according to the loss of the GAN. After iterations, with a comparable capability of the two networks, the Generator was able to successfully convert the simulated EEGs to the convincingly share the same characteristics as the real collected data.

## Experimental Data

In the experiment, the latest EEG-based control paradigm assisted by microexpressions (ME-BCI) (Zhang et al., 2021b) proposed by our team was adopted, to verify the feasibility of scalp EEG modeling and its application in BCI training.

### Experimental Electroencephalograms Acquisition

**FIGURE 9:**
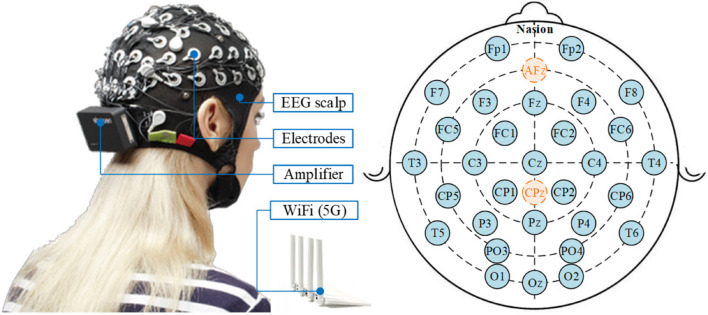
The Neuracle wireless EEG acquisition system and its electrode placement, in which AFz is the ground and Cpz is the reference by default.

The commercial wireless EEG acquisition system (Neuracle Technology Co., Ltd.) with 30 EEG channels and 1000 Hz sampling rate is adopted in this study, as illustrated in Figure 9. The electrode placement is demonstrated also in Figure 9, in accordance with the international 10-20 location system, in which AFz is the ground and Cpz is the reference by default. Eight healthy subjects (25-38 years of age) participated in the experiment (World Medical Association, 2013).

**FIGURE 10:**
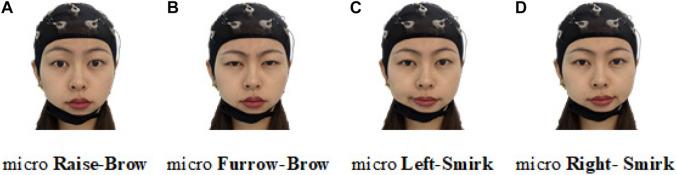
Illustration of four selected microexpressions. **(A)** Micro raise-brow. **(B)** Micro furrow-brow. **(C)** Micro left-smirk. **(D)** Micro right-smirk.

**FIGURE 11:**
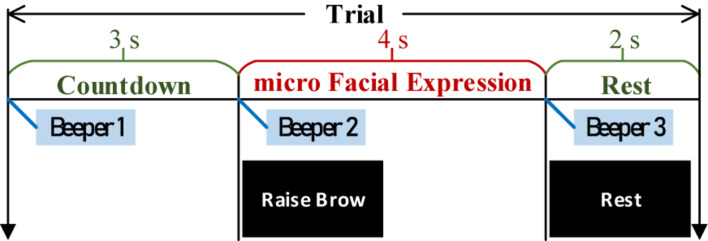
Timing diagram of each trial.

Following our latest progress on ME-BCI (Zhang et al., 2021b), real EEGs were collected. Four microexpressions were selected: micro raise-brow (mRB), micro furrow-brow (mFB), micro left-smirk (mLS), and micro right-smirk (mRS), as illustrated in Figure 10. Each microexpression was conducted for 4 sessions (including 6 trials) per subject. In each trial, 3 s countdown, 4 s microexpression, and 2 s rest were contained in turn, as demonstrated in Figure 11. During data collection, subjects were asked to sit quietly and avoid extra body movements.

In the ME-BCI related serial works (Lu et al., 2018a, 2020; Zhang et al., 2021b), for the real-time requirement, each result was decoded with 100 ms EEG input. Thus, in this work, the EEG sequences were still sliced into pieces with short-window-length. Real collected EEG data were divided into two parts: 1st session as a template for simulated EEGs conversion, 2nd ∼ 4th sessions as testing-set for feasibility verification.

### The Head Models and the Dipole Position

**FIGURE 12:**
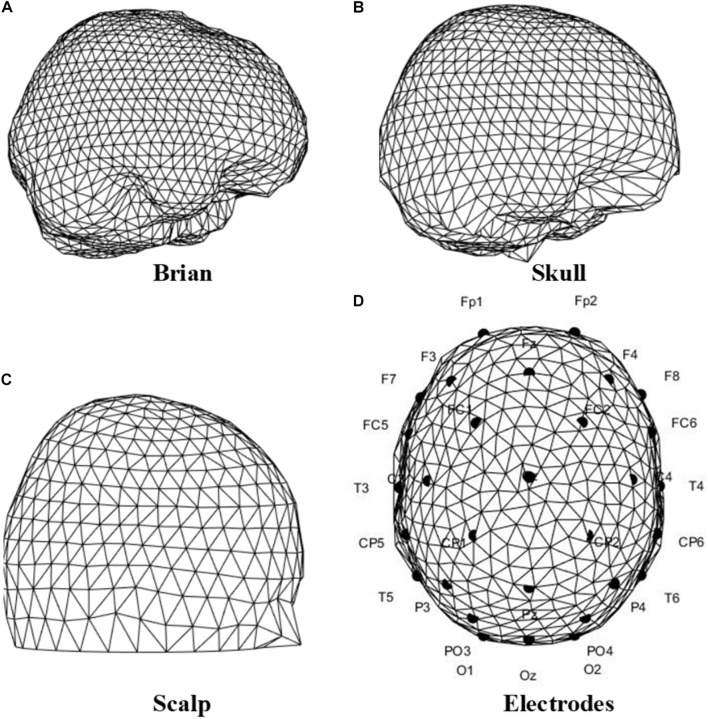
Triangulated surfaces of the standard head model, and the electrodes location of the Neuracle device. **(A)** Brain. **(B)** Skull. **(C)** Scalp. **(D)** Electrodes location according to the Neuracle device.

Facing the lack of one’s unique tissue conductivity and geometry, the three-shell standard mesh of the human head in FieldTrip (Oostenveld et al., 2010) was adopted as a template. In this standard mesh, the head structure is simplified into several surfaces, including brain, skull, and scalp; On the scalp are the EEG electrodes. Figure 12 shows the surfaces of the standard head model, and the electrodes in accordance with the Neuracle Device.

**FIGURE 13:**
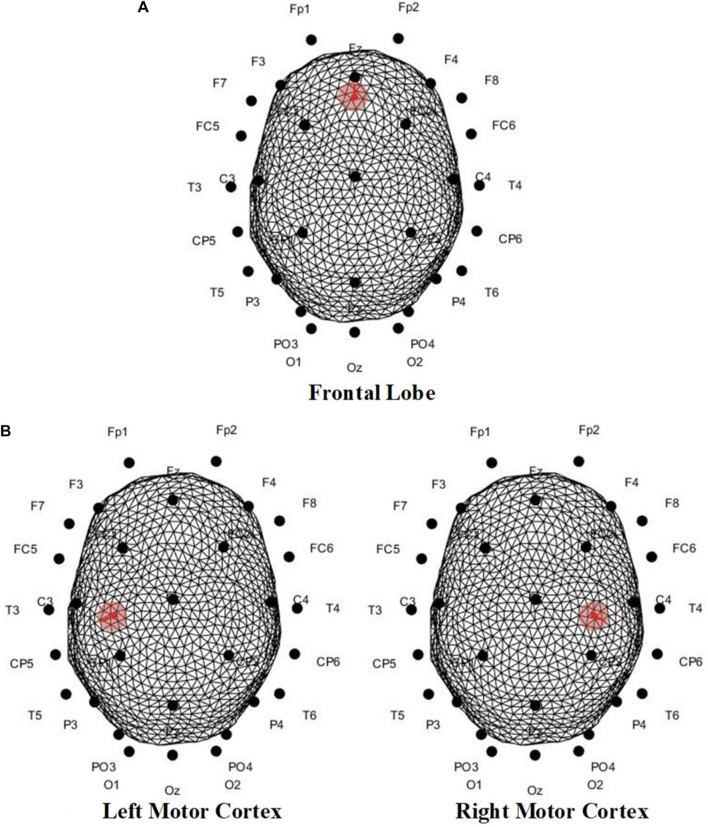
Dipole position of scalp EEGs simulation in **(A)** frontal lobe, **(B)** left motor cortex, and **(C)** right motor cortex. For mRB and mFB, dipole was set in the frontal lobe; for mLS was the right motor cortex, and for mRS was the left motor cortex.

As for the location of the EEG generator, since the precise positioning cannot be achieved without the extra professional equipment, the dipole was set in accordance with the mechanism. In our serial research on ME-BCI (Zhang et al., 2016, 2021b; Li et al., 2018b; Lu et al., 2018a, b, 2020), data-driven brain connectivity analysis demonstrated the main involvement of the motor cortex (Lu et al., 2018a; Zhang et al., 2021b), which conformed to the contralateral control facts. Meanwhile, evidence showed the frontal lobe and limbic system also participate in facial-expression processing (Price and Drevets, 2010; Li et al., 2018b; Lu et al., 2018b). Thereby, the dipole was set orthogonalization to the surface in the frontal lobe/motor cortex, separately for different microexpressions (Figure 13).

## Results

### Simulation of Multi-Channeled Scalp Electroencephalograms

#### Postsynaptic Membrane Potential

To simulate postsynaptic membrane potentials in three representative EEG bands as low (4-12 Hz), medium (12-30 Hz) and high (>30 Hz), different parameter values were taken in tuning the peaks. Part of parameters in (1) and (2) are common for each band whose values are determined according to anatomical facts (Table 4; Freeman, 1987; Jansen and Rit, 1995).

**TABLE 4:** Comman parameters in NMM.

Parameter	Value
C_1_	135
C_2_, C_7_	108
C_3_, C_4_	33.75
C_5_	40.5
C_6_	13.5
*s*_0_	6 mV
*e*_0_	2.5 s^–1^
*R*	0.56 V^–1^

**FIGURE 14:**
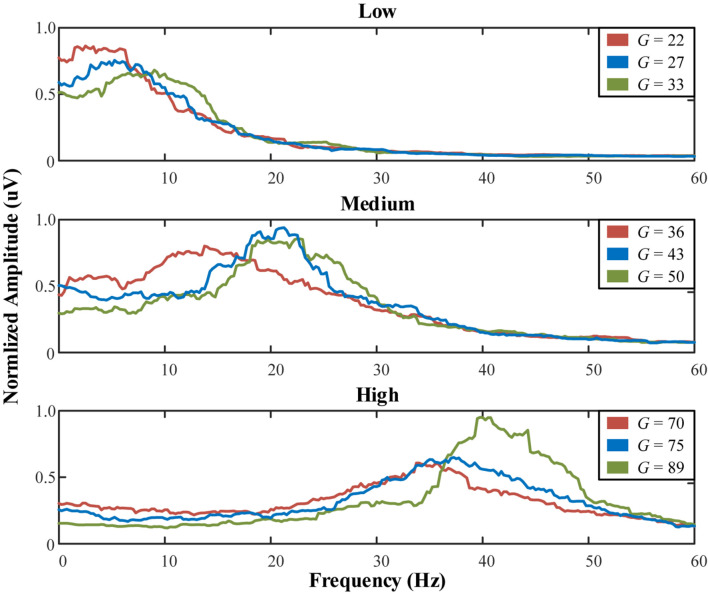
Normalized spectrum of the low, medium, and high populations with varying *G*.

Using the 4th order Runge-Kutta algorithm, differential equations in (1) can obtain accurate numerical solutions. Under certain parameter combinations, the peak can be fine-tuned by adjusting only *G* (the average gain for fast inhibitory interneurons) (Zavaglia et al., 2006). Setting the exogenous input P(*t*) as a random noise (mean value = 60, variance = 100), Table 5 lists the system parameters to generate peaks approximately in three bands. Figure 14 illustrates the spectrum of each population.

**TABLE 5:** System parameters in NMM for different EEG bands.

Parameter	EEG bands		
	Low	Medium	High
*A*	2.7	5.2	5.6
*B*	3.2	4.5	3.8
*G*	27	43	75
*a*	40	85	110
*b*	20	30	40
*g*	300	350	400

**FIGURE 15:**
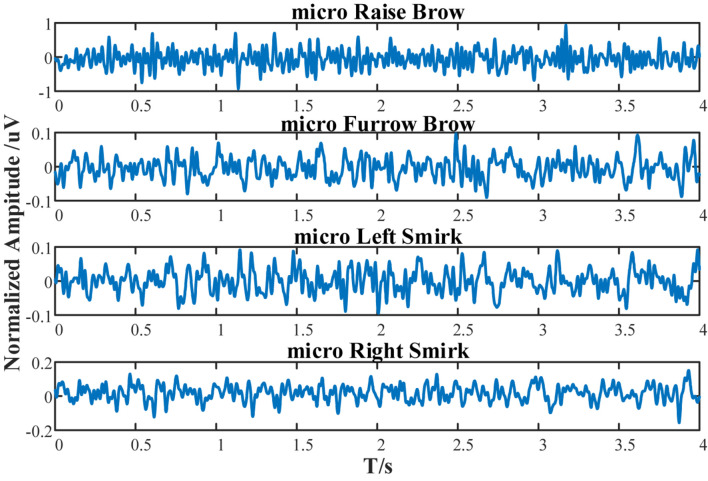
Simulated postsynaptic membrane potentials of subject S1 under micro-raise-brow, micro-furrow-brow, micro-left-smirk, and micro-right-smirk.

To mimic the subject’s EEG, population weights are calculated via one’s real EEGs (1st session of experimental data). The uniformly scaled energies within the band’s range in Real EEGs were set as the population weights, as listed in Table 6. The mimic postsynaptic membrane potentials are shown in Figure 15.

**TABLE 6:** Normalized population weights.

Microexpression	EEG Bands		
	Low	Medium	High
Raise Brow	0.1190	0.3332	0.5478
Furrow Brow	0.0845	0.0487	0.0357
Left Smirk	0.0887	0.0574	0.0467
Right Smirk	0.0996	0.0550	0.0509

#### Multi-Channelled Scalp Electroencephalograms

**FIGURE 16:**
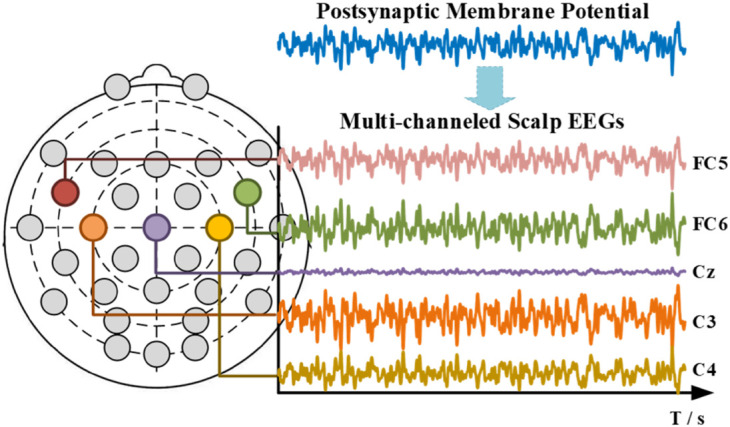
Simulated micro right-smirk multi-channeled scalp EEGs (in aesthetics consideration, only parts of channels are shown).

With the standard mesh of the human head, the forward computation was calculated with the boundary element method (BEM) for its low computational needs (Fuchs et al., 2002). According to the dipole setup, the simulated multi-channeled scalp EEGs were demonstrated in Figure 16.

**FIGURE 17:**
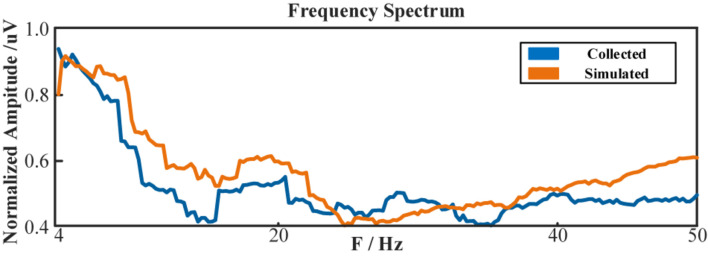
The frequency spectrum of simulated scalp EEGs and real collected data.

However, due to the blankness of the individual bio-information, the simulated multi-channeled scalp EEGs lacked realism in the temporal domain. But literature showed its capability in mimicking the spectral characteristics (Li et al., 2018a). Table 7 lists the Pearson correlation coefficient between the simulated scalp EEGs and the real collected 1st session in spectrum distribution. Figure 17 demonstrated the spectrum comparison between simulated scalp EEGs and the real collected one.

**TABLE 7:** Pearson correlation coefficient of spectrum.

Subject	Facial Expression			
	micro Raise Brow	micro Furrow Brow	micro Left Smirk	micro Right Smirk
S1	0.80	0.52	0.20	0.21
S2	0.51	0.51	0.13	0.24
S3	0.68	0.50	0.27	0.32
S4	0.64	0.51	0.26	0.13
S5	0.63	0.58	0.31	0.33
S6	0.66	0.44	0.22	0.24
S7	0.82	0.59	0.28	0.18
S8	0.66	0.55	0.22	0.27

Unlike visual evoked potentials, the frequency spectrum distribution of the ME-BCI paradigm showed no typical features (Zhang et al., 2016). As shown in Table 7, without a fine tuning in NMM parameters, dipole positions, and tissue configurations, the simulation ability of computation is limited for spectra without significant characteristics. The value in Table 7 indicated that the correlation existed, but was not high.

### Conversion and Application of Simulated Scalp Electroencephalograms

**FIGURE 18:**
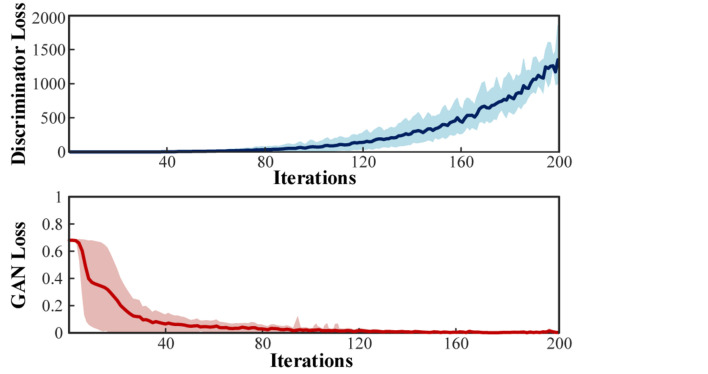
Training loss of the Discriminator and GAN.

Given the lack of biological information, the theoretical simulation of multi-channeled scalp EEGs can only reproduce some general phenomenon. To complete the conversion of simulated EEGs to its useful practical version in BCI, the simulated signals were fed into the conditional GAN. The training losses of the Discriminator and the GAN are illustrated in Figure 18.

**FIGURE 19:**
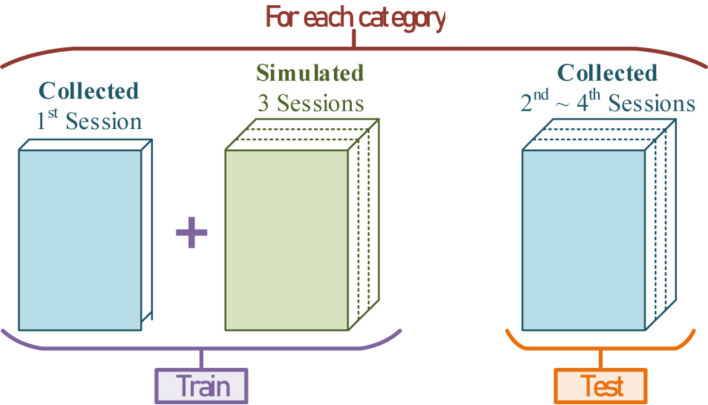
The division of training-sets and testing-sets for each category.

With the random exogenous input P(*t*), the population weights, the dipole position, and the conditional label, endless practical simulated EEGs can be generated through the conversion. To verify the feasibility and capability of this method, the simulated EEGs mixed with the real collected 1st session were used to train a ME-BCI classifier, and the rest of the 2nd to 4th sessions were used to test its performance. The division of datasets is shown in Figure 19.

All EEGs, both the real and the simulated one, were detrended and filtered into [2 Hz, 55 Hz] with 4th-order Butterworth bandpass-filter. To form an intuitive comparison, same as previous research (Lu et al., 2018a, 2020), EEGs were sliced into short-windowed (100 ms) segments, and the same feature extraction method Common Spatial Pattern (CSP) was adopted to emphasize the performance between “training with only real EEGs” and “training with fake augmented EEGs.” The spatial filter of CSP was calculated via the training-set. The ‘one versus rest’ strategy was adopted in the CSP. Various commonly used classifiers were used to calculate the validation accuracy and test accuracy. The validation and test performances under ‘training with fake augmented EEGs’ are listed in Table 8.

**TABLE 8:**
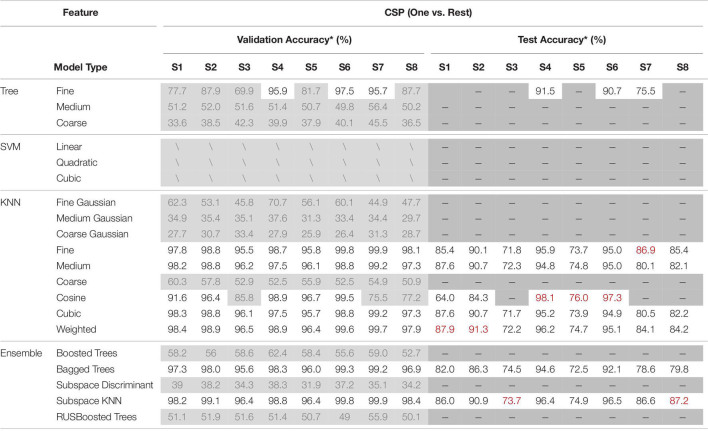
The validation and test performances under “training with fake EEGs augmented.”

*Validation accuracies were estimated via 5-fold validation, in which classifiers with training-times longer than 5 mins were not recorded and marked with ‘/’, and validation accuracies lower than 90% were marked with gray. For test accuracy, only classifiers whose validation accuracy was higher than 90% were estimated, and the highest test accuracy for each subject was marked with red.

As shown in Table 8, classifiers such as K-nearest neighbor (KNN) and some ensembles (i.e., bagged trees, and subspace KNN) achieved higher validation accuracies. As for test accuracies, due to the individual differences, the personal best varied from 73.7 to 98.1%. The mean test accuracy among classifiers is 84.93% ± 1.25. The overall performance in Table 8 indicates the feasibility of applying the simulated EEGs to the BCI classifier training, through a GAN conversion. For a detailed comparison, accuracies under the training-set with only the 1st session real EEGs were also estimated. The validation and test performances under ‘training with only the real 1st session EEGs’ are shown in Table 9.

**TABLE 9:**
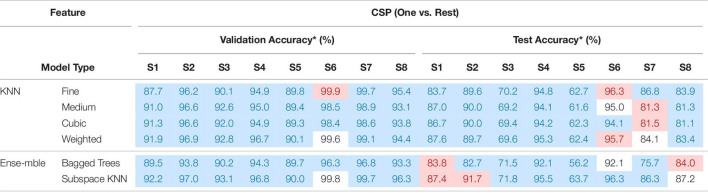
The performances under “training with the real 1st session EEGs.”

*The blue block indicates “the accuracy was improved with simulated EEGs augmented,” the red block indicates a decline, and the white block indicates an equivalence.

Comparing Table 9 with Table 8, most accuracies have been improved after the data augmentation with simulated EEGs, both the validation and the test. The two-sample *T*-test shows that after adding the simulated EEGs, the overall performance (both validation and test) has a significant improvement (*P* = 0.04 < 0.05), compared with insufficient real data. By marking the improvement in test accuracy as positive, and the decline as negative, the overall performance under augmenting with fake EEGs is 2.17% ± 4.23. Among all subjects, test accuracy of S5 achieved the largest improvement as 12.60% ± 1.81 among 6 classifiers. The comparison between Tables 8, 9 confirms the feasibility and capability of scalp EEGs simulation as a new data augmentation method in BCI.

**FIGURE 20:**
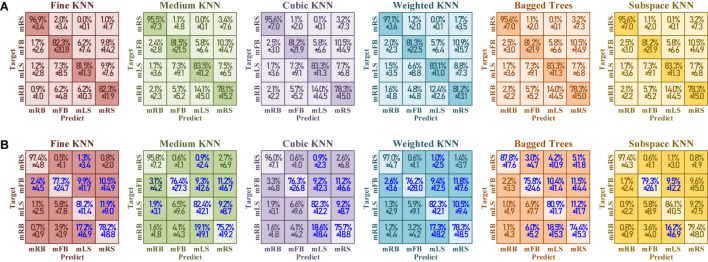
The confusion matrix of test accuracy under **(A)** training set with “fake EEGs augmented” versus **(B)** training set “only real EEGs.” The blue highlight represents that the performance has been improved with the simulation contained.

Figure 20 demonstrates the confusion matrix of six classifiers under training with “fake EEGs augmented” vs. “only real EEGs”. The blue high-lighted blocks in Figure 20B indicate that the performance has been improved after being augmented by the converted simulated EEGs. Here, the improvements mean a raise in true positive, or a decline in false positive. In all six classifiers, the distribution between “fake EEGs augmented” and “only real EEGs” basically remained the same, and showed no obvious imbalance among categories. The confusion result indicates that this training-set augmentation method with simulated EEGs shows no bias and maintains the same level of performance among categories.

## Discussion

In this work, a mathematical model of scalp EEG is presented, and a conditional GAN is put forward to convert the simulated scalp EEGs from theoretical-only to practical, hence applying in the training of BCI classifiers. Through this study, we have taken a step forward from the theoretical modeling of EEG, and initially established the connection between simulated EEGs and its practical applications in BCI. The pros and cons existing in this work are discussed as followed.

### The Significance and the Performance

#### The Tuning of Neural Mass Model Parameters, and the Structure of Coupled Neural Mass Model

Neural mass model is represented as a group of differential equations with several parameters related to physiological mechanisms. Apart from the anatomy-related common parameters (C, *e*_0_, *s*_0_, and *r*), for a single population, Jasen-Rit NMM with two feedback interneurons has four adjustable parameters (Jansen and Rit, 1995), Wendling NMM with three feedbacks has six (Wendling et al., 2002), and the more in Ursiono NMM (Ursino et al., 2010). For a multi-population model, the adjustable parameters grow exponentially, and the coupling structure also affects the output (Wendling et al., 2000). However, the peak of NMM output shows a complex non-linear relationship to the coupling of parameters. To strictly reproduce the event within collected EEGs, large efforts have to be paid or an extra fitting algorithm has to be added to tune the NMM parameters. Another difficulty in parameter tuning is the chaos of NMM (Huang et al., 2011). Even with changeless parameters, the output of NMM remains fluctuated and shows instability to different initial values and random exogenous input.

In this work, with emphasis put on the link between EEG modeling and its application in BCI, the careful selection of the NMM parameters for each individual seems unaffordable and less practical. With a GAN converter added, the strict re-produce of EEG characteristics is no longer obsessed, thereby largely ease the NMM parameter tuning step.

Since single NMM can only generate a unimodal spectrum, for cortical responses, multi-coupled NMM was adopted in the modeling of postsynaptic membrane potential. In Jansen and Rit, 1995; Zhang et al., 2016; Li et al., 2018a, a double-column model was used to model the generation of neural activity potential. In this work, the triple-population coupled NMM structure was adopted; Compared to the double-column model, the simulated output of triple-population showed more details within the EEG band, and can reproduce the fast activity within EEG. As for the studies that involve triple coupled model, such as Zavaglia et al. (2006) and Zhang et al. (2021a), different detailed population weights were set to adjust the coupling proportion within multi-populations. Such population weights were calculated via collected personal EEG data, thus improving the realism and enhancing the personal characteristics of the cortical response.

#### Overcome the Lack of Personal Brain Tissue Data

From the postsynaptic membrane potential to the scalp EEGs, personal brain tissue data matters a lot. These data include the geometric shape of the brain surface, the tissue conductivity, and the position of the cortical sources. Even by using the BEM method, further combing a distributed source model (Dale et al., 2000), the realistic cortical shape is still crucial in the forward computation. This information by far has to be determined from Magnetic Resonance Imaging (MRI). An MRI system costs millions of dollars. For non-medical department students or even some colleges, there is little chance of accessing the MRI. Even if it can be detected, this personal and costly modeling method is not practical in the field of BCI. After all, in application, it is impossible for every user to undergo MRI detection.

To overcome these obstacles we faced during research, the GAN converter was used in this work to make up for the default in personal data. Focusing on the BCI field, not only simulation but application, with the method in this work, large numbers of applicable scalp EEGs can be generated. Meanwhile, it should be made feasible to apply the simulated EEGs to the training of BCI classifier.

#### Build the Connection Between Electroencephalogram Simulation and Application in BCI

Electroencephalogram modelling in BCI area is related to specific mental tasks or BCI paradigms, but it by far has not formed guidance for the paradigm design or built any connection with its application in BCI; In other words, the EEG modelling in BCI derives from BCI but exists independently of BCI, lacking integrity. Jansen and Rit (1995) simulated the generation of visual evoked potential in EEG, which outputted signals with approximate phase response and, in detail, exported the influence of NMM parameters. But, strictly, the output ended up as the postsynaptic membrane potential, and no more properties other than phase were discussed. Zavaglia et al. (2006) reproduced the power spectral density and the temporal changes of cortical EEG during finger movement; However, the use of large fitting methods (in optimizing NMM parameters) and the MRI measurement made it difficult to establish the connection with the application in BCI. Delphine’s work (Cosandier-Rimele et al., 2010) established the computational modeling of epileptic activity, proposed a solid study in scalp EEG simulation; Although this work was unrelated to BCI paradigms, it indicated that the scalp EEG simulation are very sensitive to the geometry and electrical properties of the different head tissues (mainly, the brain, skull, and scalp), discussing the gap between the simulated-EEG and the actual-EEG and its difficulty of application. Recently, Li et al. (2018a) simulated the EEG frequency response of the scene graph steady-state visual evoked potentials; Similarly, the output ended up at the postsynaptic membrane potential, discussed no other properties besides the visual response frequency, and formed no guidance for paradigm design or application. In the latest, Hanzhe (Zhang et al., 2021a) built the mathematical model of EEG for lower limb voluntary movement intention based on NMM; However, the output did not propagate to the scalp EEG, and made no comparison with the real collected signal.

The works above did make large amounts of progress in EEG modeling related to different BCI paradigms or mental tasks, however a large number of them stopped at the postsynaptic membrane potential, and focused mainly on the reproduction of little EEG response characteristics. Compared to them, this work focused on establishing the connection between the EEG modeling and its application, by proposing the scalp EEG model and applying the simulated scalp EEG into the training of BCI classifiers, so as to add to the modeling study; The 2.17% ± 4.23 improvement of test accuracy demonstrated the feasibility. Meanwhile, in order to establish such a connection, compared to those precise modeling methods that required individual tissue data, this work adopted the standard head mesh during the simulation and proposed an additional converter to overcome such a data default problem.

### The Limitations and Further Work

#### The Overfit Problem in Classification

In the transformer from the awkward simulated EEGs to the applicable version, a conditional GAN was used. During the learning process of GAN, the signals in the real collected 1st session were used as the templates. The Discriminator would be deceived if the input showed similar features to the real templates. According to this logic, the Generator would finally output converted signals with the same features based on the judgment condition of the Discriminator. Therefore, the converted simulated signals would show distinct characteristics similar to templates.

It is widely recognized that surface EEG is a non-linear and unstable signal with a low signal-to-noise ratio, and is easily affected by one’s thinking or emotions. This attribute makes the EEG tend to show different feature distribution with time-shifting. In other words, the feature space or data distribution extracted under partial time has poor generalization. Thus, the BCI classifier would show large overfitting if trained with samples lacking diversity.

However, the simulated EEGs converted from GAN would have similar characteristics as the 1st session, so does the feature space. Since the focal point in this work is not emphasized on the EEG decoding, the feature extraction algorithm and the machine learning method are not discussed. Comparing Table 8 to Table 9, the accuracy in Table 8 shows a larger descent between the validation and the test than in Table 9. It can be found that, with the simulated EEGs added to the training, the test performance has been improved, but the over-fitting problem has been exacerbated at the same time. In future work, more attention should be paid to the diversity of the converted EEGs. The attentional mechanism can be involved (Lashgari et al., 2021), to focus more on the main characteristics so that to enable more randomness in other secondary characteristics.

#### The Lower Adaptability Among Classifiers

In Table 8, taking subject S4 as an example, among various classifiers, the validation accuracies range from 27.9 to 98.9%. For all subjects, classifiers such as “coarse tree,” “medium Gaussian SVM,” “coarse Gaussian SVM,” and “subspace discriminant” achieved low validation accuracies. While several KNNs performed better. The same result did not appear in the case of training with all real collected EEGs (Lu et al., 2020), although there are difference between real datasets.

As discussed above, the simulated EEGs converted by GAN shall appear the characteristic that accords with the discriminator’s judgment basis. According to Table 8’s result, such characteristics are more suitable with the computation logic of KNN. Further, it indicates that there are flaws in the design of the Discriminator in this article, perhaps the network structure or the cost function. Additionally, the symmetrical network structure tends to be adopted in the design of GAN, thus the same defects also exist in the Generator. In the next studies, more attention will be paid to the design of converter GAN, to ensure a better adaptability of simulated EEGs’ application among different classifiers; Additionally, more kinds of decoding algorithms will also be adopted to estimate the feasibility, such as methods with hybrid-domain features (Ieracitano et al., 2021), end-to-end deep learning methods, transfer learning methods and so on.

## Conclusion

This work established the connection from the EEG simulation to its application in BCI, and proved its feasibility in improving the test accuracy compared to insufficient real data. In this paper, a mathematical model of surface EEG was presented, and a GAN converter was proposed to transfer the theoretical simulated EEGs to its applicable version in BCI training.

In accordance with the physics of surface EEG, the mathematical computation is firstly started with a triple-population coupled NMM. Then the dipole and the forward computation were followed to model the propagation. To overcome the lack of individual biological data and build a bridge between the simulation and the application, a GAN converter was established to transfer the simulated scalp signal to its applicable version. In application, the converted simulated EEGs were used in the training of BCI classifiers. With the simulated EEGs added into the training-set, compared with only insufficient real collected data, the overall performance improved significantly (*P* = 0.04 < 0.05), and the test performance showed an overall 2.17% ± 4.23 increase. Among all subjects, the largest increase is 12.60% ± 1.81. Through this work, we hope to provide a novel feasible solution for the application of surface EEG modeling in BCI.

## Data Availability Statement

The raw data supporting the conclusions of this article will be made available by the authors, without undue reservation.

## Ethics Statement

The studies involving human participants were reviewed and approved by the institutional review board of Xi’an Jiaotong University (No. 20211452), and all experiments were conducted in accordance with the Declaration of Helsinki. The patients/participants provided their written informed consent to participate in this study. Written informed consent was obtained from the individual(s) for the publication of any potentially identifiable images or data included in this article.

## Author Contributions

XZ supervised this work and revised the manuscript. ZL proposed and did the research, and wrote the manuscript. TZ, HL, and YW organized and carried out the experiments. QT revised the manuscript. All authors contributed to the article and approved the submitted version.

## Conflict of Interest

The authors declare that the research was conducted in the absence of any commercial or financial relationships that could be construed as a potential conflict of interest.

## Publisher’s Note

All claims expressed in this article are solely those of the authors and do not necessarily represent those of their affiliated organizations, or those of the publisher, the editors and the reviewers. Any product that may be evaluated in this article, or claim that may be made by its manufacturer, is not guaranteed or endorsed by the publisher.
